# Supplementary material for: Political participation and voluntary associations : A hypergraph case study
Source: PLoS One. 2025 Jan 27;20(1):e0311626. doi: 10.1371/journal.pone.0311626 (PMC11771882; doi:10.1371/journal.pone.0311626)
Supplement: S1 File — (PDF) [file pone.0311626.s001.pdf]

Supporting information

In this section, We explain the community detection algorithm employed and compare the results obtained using two different representations: the hypergraph and its clique expansion. The community detection algorithm is based on modularity optimization, which aims to identify partitions that maximize the modularity function.

1 Modularity

This quality function was initially introduced by Newman and Girvan [1]. For a given partition of nodes, it measures the extent to which links are formed within clusters rather than between them, comparatively to a null model. For simplicity, consider an unweighted network with n nodes and m edges, endowed with an adjacency matrix $\mathbf{A}$, and a partition of nodes into q clusters $\mathcal{C} = (C_1, \ldots, C_q)$, the corresponding modularity Q is :

$$Q^{\mathcal{G}} = \frac{1}{2m} \sum_{i,j=1}^{n} (A_{ij} - P_{ij}) \delta(g_i, g_j)$$

where the P_{ij} are the probabilities in the null model that an edge exists between vertex i and j, and g_i denotes the cluster to which i is assigned. The Kronecker delta function $\delta(g_i, g_j)$ equals 1 if vertices i and j are in the same cluster, and 0 otherwise. The usual choice for the null model is a random graph where the expected degrees match the actual degrees in the original graph and the modularity is zero when all nodes are in the same cluster [1]. For graphs, satisfying this condition implies $P_{ij} = \frac{k_i k_j}{2m}$, where $k_i = \sum_j A_{ij}$ denotes the degree of node i.

Now, when dealing with hypergraphs, Barber [3] introduced a modularity function by adapting the adjacency matrix and the null model. Consider an unweighted hypergraph with an incidence matrix $\mathbf{B}$ of size $n \times g$ (also called biadjacency matrix). For the null model, he requested that the expected degrees of nodes are equal to the degrees in the actual hypergraph, with the same constraint holding for the degrees of edges when considering the dual hypergraph. Adding the constraint that the modularity is zero when all nodes and edges are in the same cluster leads to $P_{i,e} = \frac{k_i d_e}{m}$ where $k_i = \sum_e B_{ie}$ is the degree of node i, i.e., the number of edges e to which i belongs, $d_e = \sum_i B_{ie}$ is the cardinality of the edge e , i.e., the number of nodes that are within it, and $m = \sum_e \sum_i B_{ie}$. For a partition of vertices and edges into q clusters $\mathcal{C} = (C_1, \ldots, C_q)$, the corresponding modularity Q is :

$$Q^{\mathcal{H}} = \frac{1}{m} \sum_{i=1}^{n} \sum_{e=1}^{g} (B_{ie} - P_{ie}) \delta(g_i, g_e)$$

In the case of weighted graphs and hypergraphs, the entries of the adjacency and bi-adjacency matrices are no longer restricted to $\{0, 1\}$ and the features k_i, d_e, and m are computed using the same formula as above.
Let us note that a major difference between the two methods, is that the second one provides a partitioning of both nodes and edges possibly in the same cluster. In our case, clusters will be composed of agents and activities.

[1] For alternative definitions of the modularity function see [2] p 34

2 Algorithm

Modularity based-algorithms are designed to find the partition $\mathcal{C} = (C_1 \dots C_q)$ that maximizes the modularity. It is impractical to explore all possible clusterings due to the vast number of potential ways to partition the set of vertices. Among various optimization algorithm, [2, p27-38] we choose a greedy search method, known as the Louvain algorithm [4]. The algorithm begins with an initial clustering in which nodes are assigned to different communities. Then, the algorithm iterate two successive phases. In the first phase, nodes are shuffled, and each node is assigned to communities in a way that maximizes the modularity function. In the second phase, an aggregated graph is constructed where nodes represent communities, and the weights of the edges are determined based on the edges present in the initial graph. These two steps are iterated until no increase in modularity is possible.

3 Comparing clusters using the hypergraph and its clique expansion

In this section, we compare the performances of the clustering algorithms using the hypergraph and its clique expansion. One natural way to define the clique expansion of the hypergraph is to put a link between each pair of nodes i and j present in a hyperedge e. The aggregated weight for the edge (i, j) is denoted as w'_{ij}. Formally, the clique expansion of the hypergraph $\mathcal{H} = (V, E, w)$ is the graph $\mathcal{G} = (V', E', w')$ where $V' = V$, $E' = \{(i, j) \in e | e \in E\}$ and $w'_{ij} = \sum_{e:(i,j)\in e} w_e$.

Hypergraph clustering yields clusters consisting of both nodes and hyperedges, but we focus on node clusters by removing the hyperedges from the partitions obtained.

Nodes shuffling in the first phase of the algorithm introduces a stochastic effect. Stability assessment involves running both algorithms 100 times and calculating normalized mutual information[2] I_{norm} between resulting partitions. For the hypergraph, we find an average $I_{norm} = 0.941$ ($\sigma = 0.026$), while for the clique expansion, the average $I_{norm} = 0.936$ ($\sigma = 0.039$), showing stability in both cases.

The average I_{norm} between hypergraph and clique expansion partitions is 0.753, indicating similarity but not complete alignment. Now the question is where does this difference lie in our case? One possible explanation is the number of clusters and the distribution of cluster sizes $Vol(C) = \sum_{i\in C} s_i$, where s_i is the strength of agent i. Indeed, the clustering of the clique expansion graph leads to $\overline{q^{\mathcal{G}}} = 9.82$ clusters with a standard deviation of cluster size $\overline{std^{\mathcal{G}}} = 1652$ whereas the original hypergraph yields $\overline{q^{\mathcal{H}}} = 13.69$ and $\overline{std^{\mathcal{H}}} = 1123$. Hence, the hypergraph clustering provides a finer resolution and more balanced partitions.

To go into further details, we introduce the so-called *resolution* parameter γ [5] in the modularity functions :

$$Q^{\mathcal{G}} = \frac{1}{2m} \sum_{i,j=1}^{n} (A_{ij} - \gamma P_{ij}) \delta(g_i, g_j)$$

$$Q^{\mathcal{H}} = \frac{1}{m} \sum_{i=1}^{n} \sum_{e=1}^{g} (B_{ie} - \gamma P_{ie}) \delta(g_i, g_e)$$

[2] Let X and Y be two partitions, $I_{norm}(X, Y) = \frac{2I(X,Y)}{H(X)+H(Y)}$ where $I(X, Y)$ is the mutual information of X and Y and $H(X)$ is the entropy of partition X. $I(X, Y) = \sum_{x,y} \mathbb{P}(X = x, Y = y) \log \frac{\mathbb{P}(X=x,Y=y)}{\mathbb{P}(X=x)\mathbb{P}(Y=y)}$ and $H(x) = -\sum_x \mathbb{P}(X = x) \log \mathbb{P}(X = x)$. $\mathbb{P}(X = x)$ is the probability that a vertex is assigned to a community x in the scheme X. I_{norm} takes its values between 0 and 1.

Larger γ leads to more communities, while lower gamma leads to fewer communities. Now, the question is still there a difference in the resulting partitions when the number of clusters is fixed? To answer this, we perform the algorithms for a range of values of γ, and for each value of gamma, we run both of them a 100 times. Then, we compute I_{norm} for pairs of partitions having the same number of clusters while using the two different methods. Figure 1a displays the results. For values of $q \lesssim 13$, I_{norm} is relatively low and unstable. However, for $q \gtrsim 13$, I_{norm} fluctuates around 0.85.

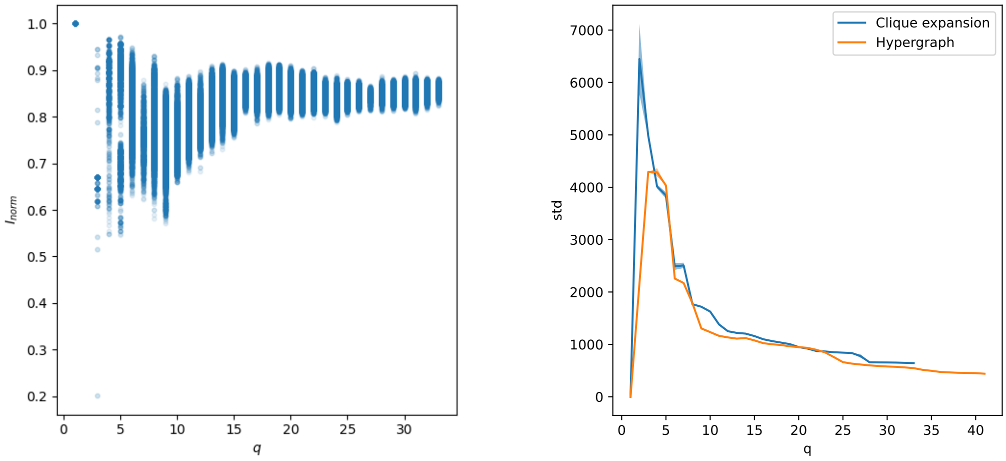

(a) (b)

Figure 1: (a) Normalized mutual information versus the number of clusters γ. (b) Standard deviation of cluster sizes versus number of clusters. The orange curve corresponds to the clustering obtained using the original hypergraph and the blue curve corresponds to the clustering obtained using its clique expansion. The colored area around the curves correspond to the standard error.

Figure 1b displays the standard deviation of clusters' size for a range of values of q, the number of clusters. The orange curve corresponds to the clustering obtained using the original hypergraph and the blue curve corresponds to the clustering obtained using its clique expansion. In both cases, for $q = 1$, all the nodes are gathered in the same cluster, leading to a vanishing standard deviation. For low values of $q \lesssim 7$, the large values of the standard deviation indicate that both algorithms provide a giant cluster that gathers most of the nodes. This tendency decreases more rapidly for hypergraph clustering. Moreover, we can note that the orange curve is almost always under the blue curve, which indicates that the hypergraph clustering provides more balanced partitions in terms of sizes.

To conclude this part, we have shown that in our case study, when using the original modularity function with the resolution parameter $\gamma = 1$, the hypergraph clustering provides a larger number of clusters and these clusters are more balanced in size. The partitions obtained with clique expansion clustering illustrate a well-known problem in modularity-based algorithms, the so-called *resolution limit* [6]. One way of solving this problem is to introduce the resolution parameter γ in the expression of the modularity function. Indeed, when we tune the resolution parameter and compare the partitions that share the same number of clusters, partitions are more similar, but the hypergraph clustering still provide more balanced partitions. Moreover, usually, we do not know the number of clusters behind the network's architecture and determining the resolution parameter that reveals the best this structure is a task on its own. Thus, in our case study, hypergraph clustering better faces the resolution limit problem than the clique expansion clustering. This empirical observation deserves to be deepened.

4 Recovering organization structures using the clustering algorithms

Figure 2 shows the distribution of similarity obtained from the hypergraph and the clique expansion clustering. For organization similarity, both inter (a) and intra (b) similarity curves are nearly identical. Concerning category similarity, while the inter-similarity (c) displays a similar distribution for both clustering methods, the intra-similarity (d) from hypergraph clustering demonstrates a higher frequency of large values and a lower frequency of small values. This indicates that the hypergraph clustering is more effective in capturing the community structure based on organization categories.

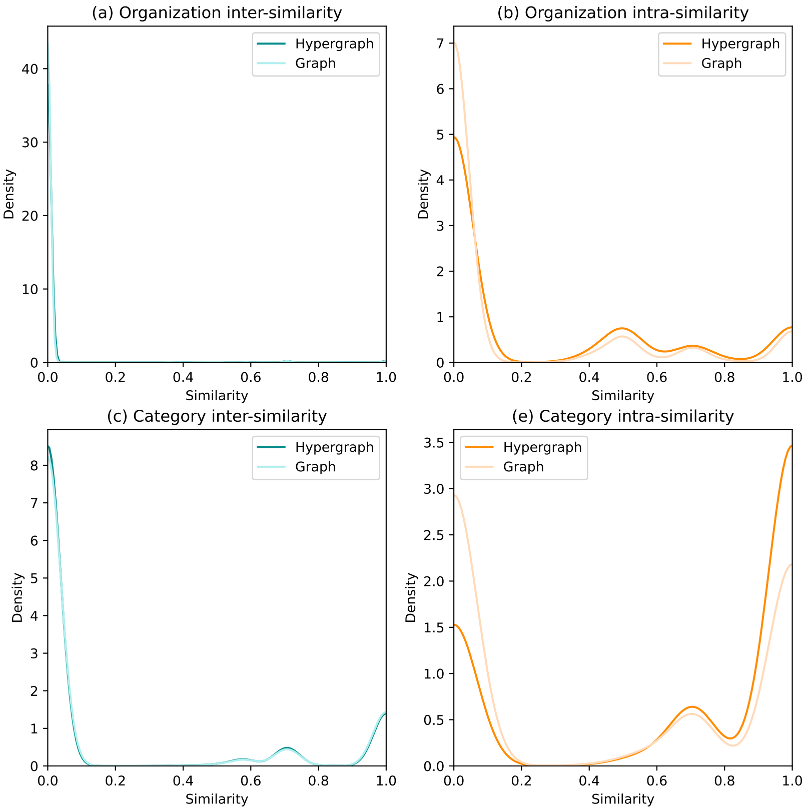

Figure 2: **Distribution of similarity with hypergraph and clique expansion clustering.** (a) Organization inter-similarity, (b) organization intra-similarity, (c) Category inter-similarity and (d) Category intra-similarity. Intra-similarity is the similarity between a pair of agents within the same cluster, while the inter-similarity corresponds to the similarity between a pair of agents belonging to different clusters. The resolution parameter is set to 1. For both hypergraph and clique expansion clustering, we run the algorithm 700 times and select the partition with the highest modularity.

References

[1] Newman MEJ, Girvan M. Finding and evaluating community structure in networks. Physical Review E. 2004;69(2):026113. doi:10.1103/PhysRevE.69.026113.

[2] Fortunato S. Community detection in graphs. Physics Reports. 2010;486(3-5):75–174. doi:10.1016/j.physrep.2009.11.002.

[3] Barber MJ. Modularity and community detection in bipartite networks. Physical Review E. 2007;76(6):066102. doi:10.1103/PhysRevE.76.066102.

[4] Blondel VD, Guillaume JL, Lambiotte R, Lefebvre E. Fast unfolding of communities in large networks. Journal of Statistical Mechanics: Theory and Experiment. 2008;2008(10):P10008. doi:10.1088/1742-5468/2008/10/P10008.

[5] Reichardt J, Bornholdt S. Statistical mechanics of community detection. Physical Review E. 2006;74(1):016110. doi:10.1103/PhysRevE.74.016110.

[6] Fortunato S, Barthélemy M. Resolution limit in community detection. Proceedings of the National Academy of Sciences. 2007;104(1):36–41. doi:10.1073/pnas.0605965104.
