# Supplementary material for: Political participation and voluntary associations : A hypergraph case study
Source: PLoS One. 2025 Jan 27;20(1):e0311626. doi: 10.1371/journal.pone.0311626 (PMC11771882; doi:10.1371/journal.pone.0311626)
Supplement: Attachment — Submitted filename: Rebutal Letter.pdf [file pone.0311626.s002.pdf]

PONE-D-23-42942
Political Participation and Voluntary Associations : A Hypergraph Case Study
PLOS ONE

Dear Dr. Azaiez,

Thank you for submitting your manuscript to PLOS ONE. After careful consideration, we feel that it has merit but does not fully meet PLOS ONE's publication criteria as it currently stands. Therefore, we invite you to submit a revised version of the manuscript that addresses the points raised during the review process.

Please submit your revised manuscript by Aug 10 2024 11:59PM. If you will need more time than this to complete your revisions, please reply to this message or contact the journal office at plosone@plos.org. When you're ready to submit your revision, log on to https://www.editorialmanager.com/pone/ and select the 'Submissions Needing Revision' folder to locate your manuscript file.

Please include the following items when submitting your revised manuscript:

- A rebuttal letter that responds to each point raised by the academic editor and reviewer(s). You should upload this letter as a separate file labeled 'Response to Reviewers'.
- A marked-up copy of your manuscript that highlights changes made to the original version. You should upload this as a separate file labeled 'Revised Manuscript with Track Changes'.
- An unmarked version of your revised paper without tracked changes. You should upload this as a separate file labeled 'Manuscript'.

If you would like to make changes to your financial disclosure, please include your updated statement in your cover letter. Guidelines for resubmitting your figure files are available below the reviewer comments at the end of this letter.

If applicable, we recommend that you deposit your laboratory protocols in protocols.io to enhance the reproducibility of your results. Protocols.io assigns your protocol its own identifier (DOI) so that it can be cited independently in the future. For instructions see: https://journals.plos.org/plosone/s/submission-guidelines#loc-laboratory-protocols. Additionally, PLOS ONE offers an option for publishing peer-reviewed Lab Protocol articles, which describe protocols hosted on protocols.io. Read more information on sharing protocols at https://plos.org/protocols?utm_medium=editorial-email&utm_source=authorletters&utm_campaign=protocols.

We look forward to receiving your revised manuscript.

Kind regards,

Hocine Cherifi
Academic Editor
PLOS ONE

Journal requirements:

When submitting your revision, we need you to address these additional requirements.

1. Please ensure that your manuscript meets PLOS ONE's style requirements, including those for file naming. The PLOS ONE style templates can be found at

https://journals.plos.org/plosone/s/file?id=wjVg/PLOSOne_formatting_sample_main_body.pdf and

https://journals.plos.org/plosone/s/file?id=ba62/PLOSOne_formatting_sample_title_authors_affiliations.pdf

2. Please update your submission to use the PLOS LaTeX template. The template and more information on our requirements for LaTeX submissions can be found at http://journals.plos.org/plosone/s/latex.

Thank you for the guidance. We have updated our submission to use the PLOS LaTeX template

3. When completing the data availability statement of the submission form, you indicated that you will make your data available on acceptance. We strongly recommend all authors decide on a data sharing plan before acceptance, as the process can be lengthy and hold up publication timelines. Please note that, though access restrictions are acceptable now, your entire data will need to be made freely accessible if your manuscript is accepted for publication. This policy applies to all data except where public deposition would breach compliance with the protocol approved by your research ethics board. If you are unable to adhere to our open data policy, please kindly revise your statement to explain your reasoning and we will seek the editor's input on an exemption. Please be assured that, once you have provided your new statement, the assessment of your exemption will not hold up the peer review process.

Thank you for your guidance. In response, we have made the data useful for our study available in a GitHub directory. The URL for accessing this data is specified in the Data Review URL section of our submission. We hope this meets the requirements for data sharing and facilitates the review process.

4. Please review your reference list to ensure that it is complete and correct. If you have cited papers that have been retracted, please include the rationale for doing so in the manuscript text, or remove these references and replace them with relevant current references. Any changes to the reference list should be mentioned in the rebuttal letter that accompanies your revised manuscript. If you need to cite a retracted article, indicate the

article’s retracted status in the References list and also include a citation and full reference for the retraction notice.

Thank you for the reminder. We have thoroughly reviewed our reference list to ensure its completeness and accuracy. In response to the reviewer comments, we have added the following reference:

6. Scheufele DA, Hardy BW, Brossard D, Waismel-Manor IS, Nisbet E. Democracy Based on Difference: Examining the Links Between Structural Heterogeneity, Heterogeneity of Discussion Networks, and Democratic Citizenship. Journal of Communication. 2006;56(4):728–753. doi:10.1111/j.1460-2466.2006.00317.x

10. McClurg SD. Social Networks and Political Participation: The Role of Social Interaction in Explaining Political Participation. Political Research Quarterly. 2003;56(4):449–464. doi:10.2307/3219806.

11. Koebel M. Les profits politiques de l’engagement associatif. Regards Sociologiques. 2000;20:165–176

12. Hamidi C. ´El´ements pour une approche interactionniste de la politisation: Engagement associatif et rapport au politique dans des associations locales issues de l’immigration. Revue fran¸caise de science politique. 2006;56(1):5. doi:10.3917/rfsp.561.0005

13. Rougier C. Tenir le politique `a distance : une capacit´e citoyenne ? Retour sur l’´echec d’une tentative d’instrumentalisation partisane d’une association de loisirs. Recherches sociologiques et anthropologiques. 2015;46(1):67–88. doi:10.4000/rsa.1388.

14. Sim´eant J. Un humanitaire “ apolitique ” ? D´emarcations, socialisations au politique et espaces de la r´ealisation de soi. In: Belin, editor. La politisation; 2003.Available from: https://hal.science/hal-03872136.

21. Ohlemacher T. Bridging People and Protest: Social Relays of Protest Groups against Low-Flying Military Jets in West Germany. Social Problems. 1996;43(2):197–218. doi:10.2307/3096998.

24. Mutz DC. The Consequences of Cross-Cutting Networks for Political Participation. American Journal of Political Science. 2002;46(4):838. doi:10.2307/3088437

25. Battiston F, Cencetti G, Iacopini I, Latora V, Lucas M, Patania A, et al. Networks beyond pairwise interactions: Structure and dynamics. Physics Reports. 2020;874:1–92. doi:10.1016/j.physrep.2020.05.00

27. Ugander J, Backstrom L, Marlow C, Kleinberg J. Structural diversity in social contagion. Proceedings of the National Academy of Sciences. 2012;109(16):5962–5966. doi:10.1073/pnas.1116502109

28. Mønsted B, Sapie˙zy´nski P, Ferrara E, Lehmann S. Evidence of complex contagion of information in social media: An experiment using Twitter bots. PLOS ONE. 2017;12(9):e0184148. doi:10.1371/journal.pone.0184148.

29. Iacopini I, Petri G, Barrat A, Latora V. Simplicial models of social contagion. Nature Communications. 2019;10(1):2485. doi:10.1038/s41467-019-10431-6.

30. Sahasrabuddhe R, Neuh¨auser L, Lambiotte R. Modelling non-linear consensus dynamics on hypergraphs. Journal of Physics: Complexity. 2021;2(2):025006. doi:10.1088/2632-072X/abcea3

32. Bastian M, Heymann S, Jacomy M. Gephi: An Open Source Software for Exploring and Manipulating Networks; 2009. Available from: http://www.aaai.org/ocs/index.php/ICWSM/09/paper/view/1

36. Matthes J, Marquart F, Sikorski CV. Like-minded and cross-cutting talk, network characteristics, and political participation online and offline: A panel study. Communications. 2021;46(1):113–126. doi:10.1515/commun-2020-2080

37. Nevers JY, Bages R. Les maires des petites communes face aux enjeux de la diversification du monde rural, une enquˆete aupr`es de 436 ´elus de Midi-Pyr´en´ees; 2008. Available from: https://shs.hal.science/halshs-00216201.

38. Malet J. Les associations, source de vitalit´e du milieu rural ?:. Pour. 2009;N°201(2):97–102. doi:10.3917/pour.201.0097.

[Note: HTML markup is below. Please do not edit.]

Reviewers' comments:

Reviewer's Responses to Questions

Comments to the Author

1. Is the manuscript technically sound, and do the data support the conclusions?

The manuscript must describe a technically sound piece of scientific research with data that supports the conclusions. Experiments must have been conducted rigorously, with appropriate controls, replication, and sample sizes. The conclusions must be drawn appropriately based on the data presented.

Reviewer #1: Yes

Reviewer #2: Yes

---

2. Has the statistical analysis been performed appropriately and rigorously?

Reviewer #1: Yes

Reviewer #2: Yes

---

3. Have the authors made all data underlying the findings in their manuscript fully available?

The PLOS Data policy requires authors to make all data underlying the findings described in their manuscript fully available without restriction, with rare exception (please refer to the Data Availability Statement in the manuscript PDF file). The data should be provided as part of the manuscript or its supporting information, or deposited to a public repository. For example, in addition to summary statistics, the data points behind means, medians and variance measures should be available. If there are restrictions on publicly sharing data—e.g. participant privacy or use of data from a third party—those must be specified.

Reviewer #1: Yes

Reviewer #2: Yes

---

4. Is the manuscript presented in an intelligible fashion and written in standard English?

PLOS ONE does not copyedit accepted manuscripts, so the language in submitted articles must be clear, correct, and unambiguous. Any typographical or grammatical errors should be corrected at revision, so please note any specific errors here.

Reviewer #1: Yes

Reviewer #2: Yes

---

5. Review Comments to the Author

Please use the space provided to explain your answers to the questions above. You may also include additional comments for the author, including concerns about dual publication, research ethics, or publication ethics. (Please upload your review as an attachment if it exceeds 20,000 characters)

Reviewer #1: The article brings in its essence a proposal that brings originality and relevance to the field. The summary is well structured and adequately presents the article and its findings. The observations to be made, in this opinion, seek to improve the reader's experience, given the relevance of the work, as well as explore the study's potential contribution to the field of understanding political phenomena, especially participation, in

democratic societies.
In the introduction, the context is clear, and the objectives of the article are presented precisely.
From a writing point of view, a small revision in long and exhaustive paragraphs is recommended, especially in the presentation of theories in section 2. In this way, it would be possible to improve readers' experience in relation to the study, understanding the origin of the study variables, and more clearly, when analyzing, the relationship with theory.

- Thank you for your positive feedback and constructive suggestions. In response, we have condensed several lengthy paragraphs in Section 2 to reduce redundancy and emphasize key results from the literature. These revisions are highlighted in blue in the marked-up version.

Furthermore, in section 2, it would be possible to make the concept of political participation clearer, given its complexity and the relationship with other concepts, which derive from its understanding.

- Thank you for highlighting this important point. We have provided a clearer definition of political participation in the Introduction section:

Indeed, this literature usually limits the study of "political participation" to voting patterns and participation rates in national elections. However, social participation and political participation can be seen as a two-way flow. The concept of "politics" can be extended to all activities that serve to organize social groups, without being limited to the act of voting. In this sense, the boundaries of politics can be expanded by taking it out of its purely professional field (Koebel 2000; Hamidi 2006; Rougier 2015). From this perspective, it is not necessary tohave an established position in political spaces to effectively mobilize political techniques. For example, techniques such as the ability to speak in public, to distribute individual tasks with authority or horizontality for the organization of collective activities, to establish strategies for seizing power or contesting the authorities instituted by democratic tools, etc., can be found in both the associative and political spheres. Beyond these technical similarities, these associations can also influence politicians at the city level through their frequent contacts with city council members, for example, when organizing social events or setting up social services such as childcare or help for the elderly. In addition, politicians may see these associations as potential channels for contacting and convincing their members, thereby becoming vehicles for electoral legitimization (Koebel 2000). Without going so far as to homogenize the two spheres (Sim´eant 2003), we define political participation as access to local politics. More formally, we measure the frequency with which people participate in activities involving local elected officials.

- We also have explained the relationship between political participation and the importance of bridging ties in subsection Related work and hypotheses:

Applying this theoretical concept to explain the level of political participation, bridging ties should enhance political knowledge and understanding, thereby increasing participation in political activities (Crenson 1978; Ohlemacher 1996; Eveland and Kleinman 2013). The results of Crenson's 1978 (1978) study in Baltimore seem to support this theory. He

compared community associations in six neighborhoods to reveal the factors behind the associations' successful operation. He found that residents with loose-knit neighborhood friendship ties were more likely tobe informed about community associations and their associations better met their interest. Another comparative study by Ohlemacher (Ohlemacher 1996) reached similar results. His analysis of the organization membership affiliations networks of two protest groups in West Germany shows that successful mobilization correlates with the presence of structural bridging links.

Moreover, Ohlemacher insists on the need for these links, as their absence in the weak mobilization group leads to network fragmentation. This result is also highlighted by Eveland and Kleinman (2013) in their study of 25 voluntary groups in a university. Comparing their general and political discussion networks, they found that political discussion networks are more likely to be broken down into subcomponents than general discussion networks.

The methodology is relevant, it is clearly exposed, as is the data analysis process. As pointed out in the article, this is the most relevant point of the work, considering that the approach has a strong relationship with practical work in the field of politics, at the same time that it advances the understanding of the creation of ties in the process of political participation, observing both citizens, from multiple sectors, as well as institutions and their roles.
As for the conclusions of the study, although very well presented and structured, it would be appropriate, as a suggestion, for a slightly greater discussion of what was observed, not only as political participation in this specific context, but also to explore some possibilities for understanding the democratic process as observed. This can inspire paths for new works and approaches in different contexts.

- In response to your suggestion for a more comprehensive discussion in the Conclusions section:

Our survey does not allow us to determine whether today's association members will be tomorrow's local elected representatives. However, we note that within the current municipality studied, several members of the municipal council have beenn or still are, involved in local associations. This observation aligns with the findings from the Nevers and Bages survey (Nevers and Bages 2008), which compares 436 towns with fewer than 2500 inhabitants in the Midi-Pyr´en´ees region and found an undeniable correlation between mayors' past involvement in associations and their subsequent rise to the presidency of the municipality. Similarly, Malet's study, which compares more than 50 communes of 500 to 800 inhabitants in 40 different French departments, indicates that "80 % of association leaders in these communes have a relationship with their municipality" (Malet 2009). These findings suggest that, beyond the professional sphere, political activity can be understood as a social activity where associations serve both as training grounds for developing political techniques and as potential springboards toward the professionalization of politics.

Reviewer #2: The theoretical contributions of the article are clear and significant. The study extends existing theories of social networks and political participation by applying them in a novel context using advanced methodological tools. The use of hypergraph analysis to capture complex, higher-order interactions in social networks is particularly innovative.

I must commend the authors for revisiting well-grounded theories, particularly Granovetter's strength of weak ties and Burt's structural holes concepts, a practice that more political scientists should adopt. I also think that the attempt to step away from self-reported data and shift to real-world data from a single municipality has the potential to capture higher-order interactions that survey designs and even traditional network analysis research may miss. The authors provide convincing evidence, particularly regarding the contribution of heterogeneous activities' participation on political participation for political participation among association members.

Thank you for your positive feedback and for recognizing the theoretical contributions and innovative aspects of our study.

That being said, I have a few misgivings and suggestions which I believe the authors could address in a revised version of the paper.

1. What was the selection criteria for the 2 organizations out of each association group?

- We have clarified the selection criteria in the Data Collection section.

We identified 23 associations that frequently offer activities in the municipality. We excluded sport and art associations mainly attended by minors and those with fewer than 4 active members. Among the remaining 17, we arbitrarily selected 10 voluntary associations, two from each of the following categories : art, educational, environmental protection, sport, social.

2. Why external motivation must be limited to neighbors and not include other external forces, such as those I see active on the news (a farmers road blockade, someone running for mayor, etc.), which I am not familiar with, but push me to do something.

- We appreciate the reviewer's valuable observation, and we have addressed this by discussing the limitation of our study that does not consider the role of external forces in the Conclusions section.

While our study emphasizes the role of social ties, we acknowledge that external forces—such as media coverage, public demonstrations, or political campaigns—can also play a crucial role in shaping political behavior. It's important to note that this study is based on correlation rather than regression analysis, which is more commonly used in surveys. Although it would be valuable to comparatively assess the impact of social ties versus other external forces, doing so would require a different approach to data collection, such as a broader survey. Our study, which involved in-depth interviews with only 23 individuals, is limited in its scope.

3. The study is limited to a single municipality in France, which with its higher than average

retirees and farming, hampers generalizations, even within the French population. It is not just how it differs in nature to Paris, , but also demographically. I think the authors can do better job in explaining that the nature of the individuals in town are not as important as their findings.

- We have added a discussion of the study's generalizability and its limitations in the Conclusions section.

Nevertheless, our work is generalizable in light of other studies conducted in different contexts (Scheufele et al. 2006; Quintelier, Stolle, and Harell 2012). While these studies consider social diversity based on religion, ethno-cultural background, and political orientations, we define social network diversity as the ability to interact with distinct communities. Regardless of its definition, social heterogeneity consistently correlates with increased political participation.

4. I understand that the explanation is driven by organizational membership ties, however, as someone who lives in the village of 3,000 people, many of the social ties within our community are based on the social connections originally created by classroom membership of our children at school, not through our vocation, art or sport or social groups. Till this day, years after my kids left home, it is the parents of my kids' peers which compose my ties within the community, the ones who inform me and activate me, and vice versa. Obviously, this may be different for the older residents of an agricultural rural community, but then we are back to generalizability.

- We have expanded the discussion of our study's limitations in the Conclusion section to acknowledge the role of other social connections.

Additionally, our focus on organizational membership ties means we did not account for other types of social connections, such as those formed through children's school activities, workplaces, or community spaces like cafés. A more comprehensive study would require surveying a larger population to capture these diverse social ties.

5. Given PLOS One's general readership, you may want to provide benefit from a more detailed explanation of the hypergraph methodology and its advantages over traditional network analysis approaches. An illustration may do wonders here.

- We are grateful to the reviewer for this suggestion, and we have expanded the explanation of the hypergraph methodology and its advantages in the Related Work and Hypothesis subsection.

Now that we have set the groundwork for the sociological conceptual framework of our study, let us emphasize the choice of using hypergraphs for modeling social interactions. In our case, face-to-face interactions correspond to individuals participating in the same activity. To model these interactions, a natural representation is a hypergraph rather than a graph since the activities under consideration possibly involve more than two individuals. Unlike traditional graphs, hypergraphs offer the possibility to represent hyperedges linking more than two nodes. Projecting this network into its clique expansion by artificially creating

links between each pair of nodes present in a given hyperedge results in an intentional loss of information.

This choice is not merely for convenience; recent studies reveal the importance of preserving higher-order interactions in various structural analyses such as centrality measurement or community detection algorithms (Battiston et al. 2020; Knoke et al. 2021). The interest in using this type of modeling is particularly significant when studying diffusion in social systems, such as the adoption of innovations or norms, the spread of rumors, and opinion dynamics. Empirical studies (Ugander et al. 2012; Mønsted et al. 2017) demonstrate that social contagion phenomena rely on higher-order interactions involving non-linear dynamics that cannot be reduced to the sum of dyadic interactions. Models for opinion dynamics also provide theoretical insights in this direction (Iacopini et al. 2019; Sahasrabuddhe, Neuhäuser, and Lambiotte 2021).

This paper aims to contribute modestly to this literature in two ways. First, we propose measures that extend the degree centrality of nodes by taking into account the composition of hyperedges. Second, in Section 6, we perform a comparative analysis of the results obtained using a community detection algorithm for the hypergraph and its clique expansion.

- we have enhanced the network visualization by including it as the first figure and providing a more detailed explanation in both the main text and figure caption.

The hypergraph visualization using a bipartite representation is shown in Fig 1. White nodes represent activities, and colored nodes represent agents. The size of each colored node corresponds to its strength, while the color indicates the agent's cluster affiliation, as determined by a clustering algorithm that we will analyze in detail later.

- We have added a comparison of the hypergraph clustering performance to clique expansion clustering in the Analysis of the Hypergraph Structure section, emphasizing the effectiveness of hypergraph clustering.

Additionally, we compare the results obtained with the hypergraph clustering to those from clustering using the clique expansion, as detailed in Section 6.4. We observe that hypergraph clustering is more effective in capturing the community structure based on the categories of organizations.

- We have provided a clearer explanation of eigenvector centrality for hypergraphs in the Centralities section:

EV log-exp The log-exp eigenvector centrality corresponds to the choices $f = id$, $\varphi(x) = \ln(x)$, $\psi(x) = \exp(x)$ *and* $g(x) = \sqrt{x}$. *This choice corresponds to a nonuniform hypergraph version of the tensor Z-eigenvector centrality for uniform hypergraphs (Tudisco and Higham 2021)*

EV max In this case, we set $\varphi = \psi = id$, $f(x) = x^{\alpha}$ *and* $g(x) = x^{1/\alpha}$ *with* $\alpha = 10$. *Based on the fact that the function* $(\sum_{i=1}^{n} \lambda_i^{\alpha})^{1/\alpha} \xrightarrow[\alpha\to\infty]{} \max_i(\lambda_i)$, *we have* $x_i \approx \max\{y_e, e : i \in e\}$. *Hence, a node is central if it is part of at least one important edge.*

- We have enhanced the discussion in the Conclusions section to further highlight the advantages of using hypergraphs in defining new centrality measures.

By considering node centrality relative to the centrality of the edges it belongs to, we can develop edge-dependent degree centralities that leverage the composition of edges. Similarly, defining node-dependent centralities for edges allows for various extensions of eigenvector centrality. Our findings indicate that the eigenvector centrality in its linear form (applied to the clique expansion of the hypergraph) does not correlate with political participation. However, when introducing non-linearity through eigenvector max centrality, which leverages the more complex structure of the hypergraph and accounts for edge cardinality, we observe a better correlation with political participation. While this last centrality is not intended to mimic social behavior, we hope that this result encourages going further in this work and investigates other sets of functions and their corresponding social notions.

6. The authors find that “people who are active, in general, and compared to others in their community, have more access to local politics”. However, given the correctional nature of this study, could it be that those who are active in one dimension (politics) become active in other dimensions (art, social, etc.), or perhaps both are driven by an external force (extroversion, for example). Is there a sub sample within your data which at the beginning of the year did not participate in politics but were drawn to it later by their ties?

- Thank you for raising the question regarding the potential overlap between political activity and other forms of community engagement. In response, we have expanded our discussion in the conclusion to acknowledge that while our survey does not track the longitudinal shift of individuals into politics, existing literature supports the notion that involvement in local associations often correlates with subsequent political engagement. We have included references to studies by Nevers and Bages, and Malet, which demonstrate this correlation.

Our survey does not allow us to determine whether today’s association members will be tomorrow’s local elected representatives. However, we note that within the current municipality studied, several members of the municipal council have been, or still are, involved in local associations. This observation aligns with the findings from the Nevers and Bages survey (Nevers and Bages 2008), which compares 436 towns with fewer than 2500 inhabitants in the Midi-Pyr´en´ees region and found an undeniable correlation between mayors’ past involvement in associations and their subsequent rise to the presidency of the municipality. Similarly, Malet’s study, which compares more than 50 communes of 500 to 800 inhabitants in 40 different French departments, indicates that ”80 % of association leaders in these communes have a relationship with their municipality” (Malet 2009). These findings suggest that, beyond the professional sphere, political activity can be understood as a social activity where associations serve both as training grounds for developing political techniques and as potential springboards toward the professionalization of politics.

7. The authors may want to check the following. The first one for its method and the second as another example of the benefits of network heterogeneity.

- Thank you for recommending these insightful references.

Like-minded and cross-cutting talk, network characteristics, and political participation online and offline: A panel study. Jörg Matthes, Franziska Marquart, Christian von Sikorski. Communications 46 (1), 113-126, 2021.

- We have incorporated this contribution into the discussion of our method in the conclusions section:

The purpose of this study is not to determine a causal relationship between people's interactional behaviour and their political participation (Quintelier, Stolle, and Harell 2012; Matthes, Marquart, and Sikorski 2021).

- Scheufele, D. A., Hardy, B. W., Brossard, D., Waismel-Manor, I. S., & Nisbet, E. (2006). Democracy Based on Difference: Examining the Links Between Structural Heterogeneity, Heterogeneity of Discussion Networks, and Democratic Citizenship. Journal of Communication, 56(4), 728–753.
- We have incorporated this contribution into our introduction

Beyond the structural importance of bridging ties, Ohlemacher (1996) argued that they are vectors for social heterogeneity, linking a variety of people with different values, experiences and socio-structural background. Even though some studies claim that network heterogeneity decreases individual political participation due to "cross-pressures" (Mutz 2002), more recent studies (Quintelier, Stolle, and Harell 2012; Scheufele et al. 2006) showed that political and social diversity foster political participation. Diverse networks and exposure to disagreement encourage individuals to reevaluate their knowledge and learn about alternative perspectives.

Quitellier et al in text introduction is missing parentheses.

- Thank you for pointing out the missing parentheses.

Throughout this revision, we have also made several changes to the article to enhance the clarity and synthesize our sesults.

- We have removed the table initially presented in Figure 2 of the first version, which showed the mean and standard deviation of strength in each cluster. Instead, we now provide the Gini coefficient of strength for the clusters.

Computing the Gini coefficient for the distribution of nodes strength within clusters, we find that small clusters with Vol(C) < 1000 have relatively low Gini coefficient (on average 0.34) indicating a more balanced distribution of node strength. In contrast, large clusters Vol(C) <

1000 exhibit a more unbalanced distribution of node strength, with an average Gini coefficient of 0.68

- We have revised the discussion on the similarity between agents belonging to the same or different clusters to clarify and simplify the analysis. This involved removing some less valuable results and synthesizing key findings. Additionally, we have replaced Figure 3 with a new version that we believe enhances the clarity of our analysis.
- We have also clarified and synthesized the analysis in the 'Correlation with Political Participation' section to provide a more cohesive and focused discussion of the findings.
- During our review process, we identified and corrected a mistake in the expression of eigenvector centrality.
- We have also updated Table 3 to include the results of the point biserial correlation with eigenvector centralities.

---

6. PLOS authors have the option to publish the peer review history of their article (what does this mean?). If published, this will include your full peer review and any attached files.

If you choose "no", your identity will remain anonymous but your review may still be made public.

Do you want your identity to be public for this peer review? For information about this choice, including consent withdrawal, please see our Privacy Policy.

Reviewer #1: No

Reviewer #2: No

[NOTE: If reviewer comments were submitted as an attachment file, they will be attached to this email and accessible via the submission site. Please log into your account, locate the manuscript record, and check for the action link "View Attachments". If this link does not appear, there are no attachment files.]

While revising your submission, please upload your figure files to the Preflight Analysis and Conversion Engine (PACE) digital diagnostic tool, https://pacev2.apexcovantage.com/. PACE helps ensure that figures meet PLOS requirements. To use PACE, you must first register as a user. Registration is free. Then, login and navigate to the UPLOAD tab, where you will find detailed instructions on how to use the tool. If you encounter any issues or have any questions when using PACE, please email PLOS at figures@plos.org. Please note that Supporting Information files do not need this step.
